# CULTURALLY ADAPTING A CREATIVITY AND WELL-BEING ARTS CURRICULUM FOR MANDARIN SPEAKING OLDER ADULTS

**DOI:** 10.1093/geroni/igae098.4147

**Published:** 2024-12-31

**Authors:** Doria Qu, Jeffrey Graupner, Renee Kroplewski, Ashley Mensik, Katherine Thompson

**Affiliations:** University of Chicago, Chicago, Illinois, United States; University of Chicago, Chicago, Illinois, United States; University of Chicago, Chicago, Illinois, United States; Loyola University Chicago, Chicago, Illinois, United States; University of Chicago, Chicago, Illinois, United States

## Abstract

Creativity Circle program is an English-language arts curriculum previously shown to reduce social isolation in older adults. This curriculum was translated, culturally adapted, implemented, and evaluated for a group of 16 Mandarin-speaking older adults over eight weeks. Modifications included incorporating Cantonese songs, Chinese poetry, and culturally significant art activities such as clay modeling of traditional items and illustrating group values using meaningful symbols. Of the 16 initial participants, 15 completed the program, with one dropout due to illness. Participants found the adapted curriculum helpful and culturally appropriate. Key qualitative findings reveal that participants prioritized intrinsic values such as happiness, health, and family over materialistic pursuits, and approaching aging and death with acceptance. Participants reported feeling less lonely after the series (Median=1, n=12; Likert: 5=Completely Agree, 1=Completely Disagree: “I feel lonely”),) when compared to the pre-series survey (Median=2, n=10though the difference did not reach significance (Mann Whitney U Test, U=85, p =.077). This study highlights the effectiveness of cultural adaptation in enhancing program relevance and participant well-being, underscoring the need for gerontological practices that respect diverse cultural perspectives. It demonstrates the feasibility of culturally adapted creative activities to engage older adults, reinforce cultural identity, and promote overall well-being in later life. Future research will focus on larger-scale implementation and quantitative assessment of impact on social isolation.

